# The Hospital Scandal at Bournemouth

**Published:** 1909-04-10

**Authors:** 


### THE HOSPITAL SCANDAL AT BOURNEMOUTH.

Bournemouth presents a lamentable spectacle so far as its hospital arrangements are at present concerned. A local war has been raging for a considerable time between the two hospitals, the Victoria at Bournemouth and the Royal Boscombe, which has produced a grave scandal. The Victoria Hospital situated at Bournemouth is quite two miles away from the Royal Boscombe Hospital and has no ground whatever on which to expand. In our issue of June 16, 1906, p. 198, we did our utmost to prevent competition and secure harmony before any steps were taken to increase the hospital buildings in connection with either of these institutions. All attempts to secure peace failed, however, and the authorities of the Victoria Hospital incurred the grave responsibility of acquiring a site two miles away from their institution but quite close to the Boscombe Hospital and proceeding to erect thereon an out-patient department. The history of hospitals in this country presents no more lamentable instance of defective judgment in hospital affairs than this action of the Victoria Hospital authorities. The Royal Boscombe Hospital with an adequate site were arranging for the erection of an out-patient department at the time, and they have succeeded in opening this department first. We propose to give the plans of the buildings in due course, but may here mention that we are informed that the out-patient department of the Royal Boscombe Hospital is large enough to contain the whole of the Victoria Hospital buildings with room to spare. The whole proceedings in regard to these out-patient departments is very regrettable, and it remains to be seen whether the inhabitants of the district, who will have to supply the money for doing the work twice over, will mark their sense of the action of those who are to blame, by withholding supplies. In the interests of sound hospital administration it is to be hoped that they will show this measure of intelligence in self-defence.

